# A Systematic Survey of Expression and Function of Zebrafish *frizzled* Genes

**DOI:** 10.1371/journal.pone.0054833

**Published:** 2013-01-22

**Authors:** Masataka Nikaido, Edward W. P. Law, Robert N. Kelsh

**Affiliations:** Department of Biology and Biochemistry, University of Bath, Claverton Down, United Kingdom; Texas A & M University, United States of America

## Abstract

Wnt signaling is crucial for the regulation of numerous processes in development. Consistent with this, the gene families for both the ligands (Wnts) and receptors (Frizzleds) are very large. Surprisingly, while we have a reasonable understanding of the Wnt ligands likely to mediate specific Wnt-dependent processes, the corresponding receptors usually remain to be elucidated. Taking advantage of the zebrafish model's excellent genomic and genetic properties, we undertook a comprehensive analysis of the expression patterns of *frizzled* (*fzd*) genes in zebrafish. To explore their functions, we focused on testing their requirement in several developmental events known to be regulated by Wnt signaling, convergent extension movements of gastrulation, neural crest induction, and melanocyte specification. We found fourteen distinct *fzd* genes in the zebrafish genome. Systematic analysis of their expression patterns between 1-somite and 30 hours post-fertilization revealed complex, dynamic and overlapping expression patterns. This analysis demonstrated that only *fzd3a, fzd9b,* and *fzd10* are expressed in the dorsal neural tube at stages corresponding to the timing of melanocyte specification. Surprisingly, however, morpholino knockdown of these, alone or in combination, gave no indication of reduction of melanocytes, suggesting the important involvement of untested *fzds* or another type of Wnt receptor in this process. Likewise, we found only *fzd7b* and *fzd10* expressed at the border of the neural plate at stages appropriate for neural crest induction. However, neural crest markers were not reduced by knockdown of these receptors. Instead, these morpholino knockdown studies showed that *fzd7a* and *fzd7b* work co-operatively to regulate convergent extension movement during gastrulation. Furthermore, we show that the two *fzd7* genes function together with *fzd10* to regulate epiboly movements and mesoderm differentiation.

## Introduction

During development a small number of types of signaling factors are utilized repeatedly to allow this complex and beautiful process to unfold. These signals are usually transduced by members of an equally small number of receptor molecule families, raising major questions as to how distinct responses can be generated by the same limited number of signals, and in what ways signals generated by individual members of the receptor families may be different. The Wnt family of signaling proteins comprises a key example of these signals, playing diverse roles throughout all stages of vertebrate and invertebrate embryonic development [1], [2]. The Wnt family proteins are cysteine-rich secreted glycoproteins, and they have been shown to be crucial for a variety of developmental events, such as formation of ventral mesoderm during gastrulation [3], [4], posteriorisation of neural plate [5], [6], induction of neural crest cells (NCCs) from ectoderm, and then melanocyte specification from these NCCs [7]. Wnt signaling is generally thought to be transduced principally by seven-path transmembrane proteins of the Frizzled (Fzd) family and their co-receptors, low-density lipoprotein receptor-related protein (LRP) 5, 6, with the Wnt ligands interacting with a cysteine rich domain (CRD) in the extracellular part of the Fzd protein [2], [8]. Thus it is vital that we characterize the specific Wnt and Fzd proteins associated with each of the developmental processes associated with Wnt signaling, as a crucial starting point from which to begin to understand the basis for the specific responses triggered in each case. Surprisingly, however, our knowledge of the expression patterns and functions of the *fzd* gene family remains limited to only a small subset of genes and of developmental roles [9], [10]. Therefore, we wanted to carry out a comprehensive analysis of *fzd* gene expression patterns with respect to some of the early developmental roles for Wnt signaling. To allow comprehensive assessment of the entire complement of *fzd* genes we chose to use the zebrafish model system: The centralized repository of gene and genomic information (ZFIN, http://zfin.org/; Ensembl, http://www.ensembl.org/Danio_rerio/Info/Index) allowed rapid identification of the full complement of *fzd* genes. After examining the expression patterns, the well-established method of single or multiple gene knockdown using morpholino oligos [11] allowed us to explore gene functions, including allowing for the expected functional redundancies. Such a comprehensive survey of expression and functional analysis would be expected to provide crucial information allowing more focused investigations of Wnt signaling for specific functions.

For this approach, we chose to focus functionally on three important developmental roles of Wnt signaling, which we explored as test cases; melanocyte specification, NCC induction and convergent extension (CE) movements during gastrulation. NCCs form a transient embryonic structure, and are remarkable for producing many distinct cell types, including pigment cells, peripheral neurons, glial cells, and jaw cartilage [12]. Among NCC-derivatives, melanocytes have been extensively studied because their characteristic color and morphology makes them highly amenable to genetic screening [13], [14]. Previous work has identified a conserved role for Wnt signaling in specification of melanocytes from NCCs [15]. This system thus provides a paradigm for understanding the role of Wnt signals in fate specification from multipotent progenitor (stem) cells [16], [17]. Activation of canonical Wnt signaling by injection into zebrafish embryos of mRNA encoding an activated form of ß-Catenin results in NCCs adopting melanocyte fates at the expense of the neuronal and glial lineages [17], [18]. Conversely, conditional knockout of ß*-Catenin* in mice also showed the requirement of Wnt signaling for formation of melanocytes and their precursors [19], [20]. Analysis of the expression patterns of various Wnt ligands suggests *Wnt1, Wnt3, Wnt3a* and *Wnt4* are all expressed in largely overlapping regions in the dorsal midline of neural tube [18], [21]. The lack of melanoblast marker gene expression after combinatorial knockout of *Wnt1* and *Wnt3a* in mice [22] supports the idea that *Wnt1* and *Wnt3a* are primary ligands promoting melanocyte specification. In contrast, the relevant Wnt receptor genes remain unknown.

In addition, Wnt signaling has a crucial role in NCC induction [7], [8]. NCCs, like placodal cells, are formed at the border of neuroectoderm and non-neuroectoderm early in neural tube formation. Analysis using several model organisms demonstrates that activation of Wnt signaling combined with attenuation of bone morphogenetic protein (BMP) signaling is important to induce NCCs instead of placodal cells [23], [24], [25]. In the zebrafish, Wnt8 has been shown to be a major ligand required for NCC induction [26]. *fzd7* has been identified as a key receptor required for NCC induction in *Xenopus* [27]. However, the *fzd7* gene is also required for cell movement and polarity in vertebrate development [28], suggesting the possibility that other *fzd* genes may be involved in NCC development and may provide the specificity.

Finally, non-canonical Wnt signaling plays a pivotal role in CE movements [29], [30], [31]. During gastrulation, cells that give rise to the ectoderm, mesoderm and endoderm undergo coordinated movements towards the dorsal side (convergence), and those cells intercalate around the dorsal midline resulting in elongation of the embryo along its antero-posterior body axis (extension). In contrast to our detailed understanding of the genes required for specification of cell types along the dorsoventral and anteroposterior axes, those controlling this coordinated movement remain poorly understood. Furthermore, as Wnt components required for CE movements are thought to contribute to the invasiveness of cancer tissues [32], identification of the molecular mechanisms underlying Wnt signaling in this process may well be informative for understanding the molecular mechanisms underlying cancer cell invasion and metastasis.

Comprehensive expression analysis of *fzd* gene expression would be expected to identify candidate *fzd* genes relevant to each of these developmental roles of Wnt signaling. Here, we carried out in situ hybridization analysis for thirteen *fzd* genes at multiple developmental stages relevant to the three developmental processes selected, and identified *fzd* genes that were candidates for mediating each of them. Morpholino knockdown of these identify Fzd7a, Fzd7b and Fzd10 as key receptors mediating Wnt signaling in CE movements and mesoderm differentiation. Surprisingly, morpholino-mediated knockdown, alone or in combination, of all the *fzds* considered to be candidates for melanocyte specification and NCC induction, failed to generate the expected phenotypes, suggesting the importance of ubiquitously or low level expressed *fzd* genes in mediating these processes.

## Materials and Methods

### Fish husbandry

Wild type (AB) strain and TOP:dGFP transgenic line (kindly provided by Dr. Steve Wilson) were maintained under a light and dark cycle of 14 and 10 hours, respectively. Embryos collected from natural spawnings were cultured at 28.5°C, and staged according to Kimmel et al [33]. Embryos for in situ hybridization were treated with 0.003% 1-phenyl-2-thiourea from 10 hpf (hours post-fertilization) to prevent pigmentation. The *sox10^m618^* strain was maintained as heterozygous carriers [14].

### Cloning of *fzd* genes

The primer sequences used for cloning are shown in Table 1. cDNAs for cloning were prepared from 24 hpf wild type AB embryos using TRI reagent (Sigma) and superscript III (Invitrogen). To amplify specific fragments by polymerase chain reaction (PCR), we used GoTaq polymerase (Promega); amplified fragments were cloned into pGEM-T easy vector (Promega) by standard TA-cloning. Cloned fragments were identified by restriction digestion and partial sequencing. All cloned *fzd* genes showed >98% nucleotide identity to the reported sequences, although *fzd2* showed a 38 bp deletion in the 3′UTR and is likely to represent a splicing variant. We further validated our clones by comparison of expression patterns with those reported for zebrafish and/or other vertebrates.

**Table 1:** Sequence information of *fzd* examined.

Name of gene	Sequence of forward primer	Sequence of reverse primer	Enzyme for probe	Accession No.
*fzd1*	5′-AGC TCT GCG TGG GAC AGA AC	5′-ACG AAA GCT GAG CTT CAC AC	PstI/T7	BC163358
*fzd2*	5′-CGC ATC CGA ACC ATC ATG AA	5′-AGC AAA TGA GAG AGA GTG GC	PstI/T7	NM_131140
*fzd3a*	5′-ATG AGCAGA GCC ATA GAT CG	5′-ACC CCA GAC TTC TGT TGG TA	NcoI/SP6	NM_001042761
*fzd3b*	5′-ACT GTG ACG AGT CTT ACC CT	5′-TTA CGC ACT GGT CCC GTT CT	NcoI/SP6	AB246777
*fzd4*	5′-TAA ACT GCA GCC TTT TCC CG	5′-CAC AAC CGT CTC GTT TCC TT	NdeI/T7	XM_002664725
*fzd5*	5′-TGC ATG GAC AGA AAC AGC AG	5′-TCA GAC ATG TGA TGA GGG TG	SpeI/T7	AF117387
*fzd6*	5′-TAA ATG GAT TAA GCT CCG CC	5′-GAC AGA AAC TCT GAG TTT AGA G	SalI/T7	NM_200561
*fzd7a*	5′-CAG AAC CAT CAT GAA GCA CG	5′-CAG CGA AAC TGT ACA GAT AC	SpeI/T7	AF336123
*fzd8a*	5′-AGG AGT GTC ATT AAG CAG GG	5′-GCT TTT CGT GTG GCA TCT AC	NcoI/SP6	AF039412
*fzd9a*	5′-GTA TTC CTG CAG TGA AGA CC	5′-GTT TTA TTG CTC TTC CTT TGA G	SalI/T7	NC_007116
*fzd9b*	5′-CCC ACC AGA AAT GAC CCT AA	5′-TCA TAC ATG TGT GGG ACA GT	SpeI/T7	AF169639
*fzd10*	5′-CGT AAG GTC ATG AAG ACC GA	5′-AAC CAG GGA CAT GAT CTCAC	NcoI/SP6	AF039411

### Probe synthesis and whole mount in situ hybridization

The enzymes used for making probes are also in Table 1. *fzd7b* cDNA in pSPORTvector was kindly provided by Dr. Vladimir Korzh [28]. It was linearized and transcribed with *Sal* I and SP6, respectively. Whole mount in situ hybridization was carried out as described before [34]. All embryos were cleared by transferring through a series of glycerol buffered by PBS (phosphate buffered saline). Images were taken under compound microscope (NIKON Eclipse E800) using a Digital Sight DS-U1 camera (Nikon). Image processing was carried out using Photoshop (Adobe).

### Morpholino injection

Sequences of all morpholinos used in these experiments are in Table 2. Morpholinos against *fzd9b* and *fzd10* were designed for this work (GeneTools LLC.). For *fzd3a-*MO, we used the previously published sequence [35], while for *fzd7a* and *fzd7b* we obtained sequence information for effective MOs from Dr. Vladimir Korzh (pers. comm.). As negative controls, we used either standard morpholino (CCTCTTACCTCAGTTACAATTTATA) or random control oligo 25-N provided by GeneTools LLC as noted. Morpholinos were suspended in 1xDanieau's solution (58 mM NaCl, 0.7 mM KCl, 0.4 mM MgSO_4_, 0.6 mM Ca(NO_3_), 2.5 mM HEPES, pH 7.6), and injected into yolk at 1–4 cell stages. Embryos were cultured until appropriate stages at 28.5°C, and fixed for in situ analysis. The *fzd3a-*MO used here was designed to disrupt the splicing out the first intron (16 kb) of the *fzd3a* transcript, which allowed us to assess the efficacy of this morpholino simply by reverse transcription (RT)-PCR assay as previously reported [35]. Briefly, embryos injected with *fzd3a-*MO were collected at 24 hpf, and total RNAs were extracted with TRI reagent (Sigma) for reverse transcription using Superscript III (Invitrogen) and following primers designed against the two *fzd3a* exons flanking the first intron as reported [35]; forward: 5′-ATGCTGACTGTATGCATGGCC-3′, reverse: 5′-GCCATAATCCCGTTGAACTGC-3′. The size of the normal *fzd3a* transcript amplified with these primers should be 465 bp. Under our PCR conditions, this PCR fragment cannot be detected in the experimental group due to the length of the first intron (16 kb), but is readily seen when splicing occurs normally. As a positive control for RT-PCR reaction, we used primers to amplify *β-actin* cDNA (Forward: 5′-TACCCCATTGAGCACGGTAT, reverse: 5′-GTTCCCATCTCCTGCTCAAA).

**Table 2:** Sequence of morpholinos.

Name of gene	Sequence
*fzd3a-MO*	5′-CAATGTGAATTGGTTTACCTCCATG-3′
*fzd7a*-MO	5′-ATAAACCAACAAAAACCTCCTCGTC-3′
*fzd7b*-MO	5′-TCGGCTTGTGCTTCGCTGCTATTCC-3′
*fzd9b-*MO	5′-TCACAATTTGAGGTGAGCTTCCCAT-3′
*fzd10*-MO	5′-TGAGTCCGACACCGGCAGCAAACAT-3′

### Ethics statement

This study was performed with the approval of the University of Bath ethics committee and in full accordance with the Animals (Scientific Procedures) Act 1986 under Home Office license PPL30/2415.

## Results

### Identification of the full complement of zebrafish *fzd* genes

To allow a comprehensive analysis of expression patterns of *fzd* genes, we utilized two databases, ZFIN (http://zfin.org/) and Ensembl (http://www.ensembl.org/Danio_rerio/Info/Index). At the start of this project, twelve *fzd* genes were already registered with their cDNA sequence in ZFIN; these *fzd* genes were as follows; *fzd1*, *fzd2*, *fzd3a, fzd3b, fzd4, fzd5, fzd7a, fzd7b, fzd8a, fzd8b, fzd9b,* and *fzd10* (See Table 1 for accession numbers of sequences used here). Furthermore, using the full coding sequence of reported *fzds* or cDNA sequences corresponding to their cysteine rich domain (CRD) when full coding sequence was not available, we screened for *fzd*-related genes in the zebrafish genomic DNA database in Ensembl (Zv8); a repeat of this procedure in the Zv9 release of the zebrafish genome identified the same genes. Most of those we identified were already reported in ZFIN, but *fzd9a* was newly found (see Table 1 for accession number.). Also, the *fzd6* gene was published during the course of this survey [36]. Thus, we identified orthologues for all of *fzd1-fzd10*, representing the full complement of *fzds* known from other vertebrates. We cloned sub-regions suitable for making probes for all these genes to examine their expression patterns in embryos, with the one exception of *fzd8b* which has been clearly shown to have expression in a very tightly localized pattern in the forebrain at 1-somite stage and later in the neurohypophysis [37]). Primers for cloning cDNAs were designed to amplify as long a region as possible of the 3′UTR to ensure gene specificity of probes (See Table 1 for sequences of primers used). When information for the 3′UTR was limited or unavailable, we designed primers to clone a 1–1.5 kb fragment encompassing the 3′ region of the cDNAs (See Figure S1 for location of each probe.). We prepared antisense riboprobes from these cloned partial cDNAs, and performed whole mount in situ hybridization analysis on wild-type embryos at three different stages encompassing the key stages for NCC induction and melanocyte specification. Firstly, we examined 1-somite stage embryos since this is when early NCC markers are first seen mainly in the lateral border of the neural plate [38], [39]. We expected that *fzd* genes relevant to the induction of NCC would be expressed in part or all of the ectoderm in the neural plate, prospective epidermis or both. Secondly, melanocyte specification is first detected as induction of *mitfa* expression, and begins around 18 hpf [40]. At this stage, *sox10*, which plays a key role in specification of melanocytes by direct activation of *mitfa* [41] is expressed uniformly in NCCs after delamination (premigratory NCCs) and, more posteriorly (i.e. in developmentally younger cells), in dorsal neural tube cells (NCCs prior to delamination) [38]. Consequently, we expected *fzd* genes driving melanocyte specification would be expressed in dorsal neural tube or premigratory NCCs at 18 hpf. We also examined embryos at 30 hpf when melanoblasts are readily identified among migrating NCCs on both the medial and lateral migration pathways.

**Figure 1:**
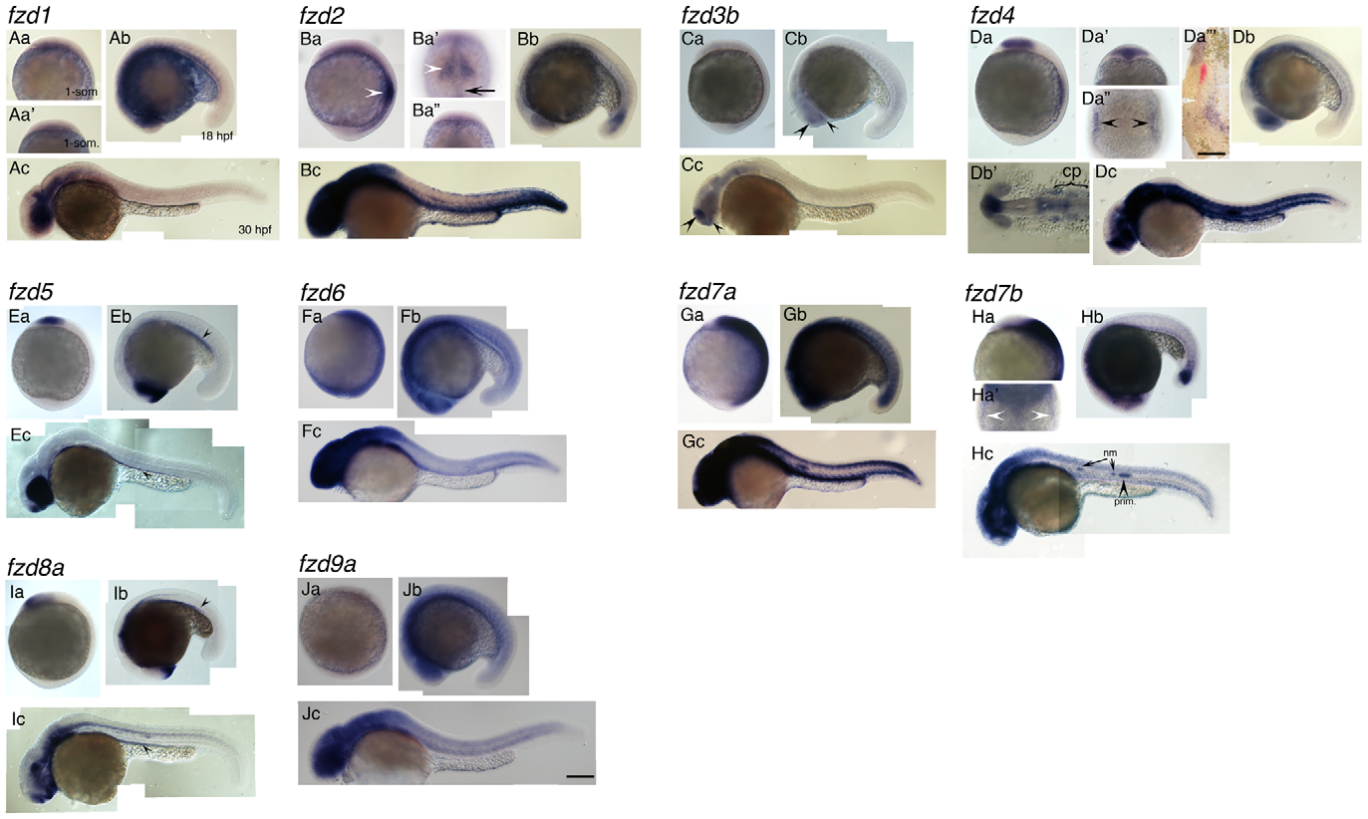
Expression patterns of *fzd* genes examined in this study. Name of each gene is indicated at top left corner. Panels Aa, Ba, Ca, Da, Ea, Fa, Ga, Ha, Ia, Ja are left side views at 1-somite stage, anterior to the top. Panels Aa', Ba', Ba'', Da', Da'', Ha' are dorsal views with anterior to the top. Panels Ab, Bb, Cb, Db, Eb, Fb, Gb, Hb, Ib, Jb are left side views at 18 hpf stage, dorsal to the top. Panels Ac, Bc, Cc, Dc, Ec, Fc, Gc, Hc, Ic, Jc are left side views at 30 hpf, dorsal to the top. Da''' and Db' are flat mounted view of *fzd4* at 1-som. and18 hpf, respectively. Anterior to the top for D''', and to the left for Db'. Da''' shows only right half of the embryo. The left edge of the embryo corresponds to the midline. For descriptions, see main text. All images are taken at the same magnification. Abbreviations: cp, cranial placode; nm, neuromast; prim., posterior lateral line primordia. Scale bar in Da''' is 200 µm, and one in Jc and for all is 100 µm.

**Figure 2:**
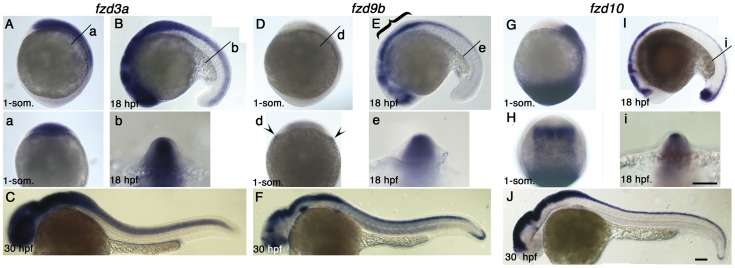
Expression pattern of *fzd3a, fzd9b* and *fzd10* at 1-som, 18 hpf and 30 hpf stages. Name of genes are indicated on top. Panels A, D, G are left side views at 1-som. stage. Dorsal to the right. Panels B, E, I are left side views at 18 hpf stage. Dorsal to the top. Panels C, F, J are left side views at 30 hpf with anterior toward left. Panels a, b, d, e, i are optical sections at the indicated level of panels A, B, D, E, I, respectively. Panel H is dorsal view with anterior set toward top. For descriptions, see text. Arrowheads in panel d indicate expression at the edge of neural plate, but these signals seem to be underneath the ectoderm. Scale bar: 100 µm.

All *fzd* genes showed specific expression patterns at one or more of the stages examined (Fig. 1 and Fig. 2). *fzd3a, fzd7b*, *fzd9b* and *fzd10* were all expressed in regions consistent with possible roles in NCC induction or melanocyte specification; three candidates for melanocyte specification (*fzd3a, fzd9b, fzd10*) will be described in detail in the next section. *fzd7b* is expressed in a pattern consistent with potential involvement in NCC induction, and will be described later in this section. Based on the expression patterns we observed, the other genes were considered highly unlikely to have roles in the processes of development that form the focus of this paper, nevertheless the expression patterns of *fzd1, fzd2, fzd3b, fzd4, fzd5* and *fzd8a* will be described briefly (Fig. 1A, 1B, 1C, 1D, 1E, 1I, respectively). We found ubiquitous expression of some *fzd* genes at 1-somite (*fzd6*, Fig. 1Fa; *fzd7a*, Fig. 1Ga) and 18 hpf (*fzd6*, Fig. 1Fb; *fzd7a*, Fig. 1Gb, *fzd9a*, Fig. 1Jb) stages, but no sign of specific expression of any of these genes in early melanoblasts in the 30 hpf stage (Fig. 1Fc, 1Gc, 1Jc); again, these were considered unlikely to be important in NCC formation or melanocyte specification (see Discussion).

*fzd1* did not show expression at 1-som. stage (Fig. 1Aa, 1Aa') whilst expression in the central nervous system (CNS) started to appear from 18 hpf (Fig. 1Ab, 1Ac). As has been described before [42], *fzd2* was expressed in the forming somite at 1-somite stage (white arrowhead in Fig. 1Ba). In addition, we found *fzd2* expression in the fin and dorsal neural tube at 30 hpf (Fig. 1Bc), although there was no expression in the trunk consistent with migrating NCCs. *fzd3b* showed significant expression in a dorsal part of telencephalon and diencephalon at 18 and 30 hpf (arrowheads in Fig. 1Cb, Cc). *fzd4* was expressed strongly as bilateral stripes located around the border of neural plate (arrowheads in Fig. 1Da”). Based on the expression pattern at 18 hpf (Fig. 1Db'), we interpreted these as marking future cranial placodes instead of NCCs. To test this idea, we performed two color in situ hybridization using probes for *fzd4* and *foxd3*, an early marker for NCC, at 1-somite stage as shown in Fig. 1Da'''. *fzd4* expression (purple) was clearly expressed in a region lateral to the *foxd3* expression domain (red), confirming that *fzd4* is not expressed in NCC, and is indeed likely to be expressed in the cranial placodes. *fzd5* expression is highly restricted to eye (Fig. 1Eb, 1Ec), forebrain (Fig. 1Ea-c) and gut (arrowheads in Fig. 1Eb, 1Ec) during development. *fzd8a* expression was seen in forebrain (Fig. 1Ia-c) and gut (arrrowheads in Fig. 1Ib, 1Ic), with strong expression becoming more widespread in the ventral CNS by 30 hpf (Fig. 1Ic).

*fzd7b* showed faint expression around the border of the neural plate in addition to strong expression in the future mid- and hindbrain at 1-somite stage (Fig. 1Ha, Ha'). It is possible that this bilateral expression pattern simply marks the future cranial placode area like *fzd4*. However, we chose the *fzd7b* gene as a candidate for further analysis of NCC induction because it is reported that the *fzd7* gene is required for NCC induction in *Xenopus* [27], and our two color in situ hybridization showed that *fzd7b* expression in the mid-and hindbrain region overlaps the *foxd3* expression domain, marking NCC (Figure S2). Taken together, we concluded that *fzd7b* was a good candidate for a role in NCC induction. Therefore, we explored this hypothesis in more detail (see later).

### Expression patterns of *fzd3a, fzd9b, fzd10* at NCC induction and pigment cell formation stages

Unexpectedly, we were unable to detect expression of any of the *fzd* genes in trunk NCCs, including differentiating melanocytes and melanoblasts, at 30 hpf (Fig. 1 and Fig. 2). Nevertheless, three genes, *fzd3a, fzd9b* and *fzd10*, showed expression patterns that included some or all of the dorsal neural tube at 18 hpf (Figs. 2B, 2E, 2I, 2b, 2e, 2i), a place where melanocyte specification is likely to occur. These genes showed distinct patterns (Fig. 2).

*fzd3a* expression in the neural plate is first detected in an anterior domain at 1-somite stage (Fig. 2A). Optical sections at the level of the posterior hindbrain (level labeled with “a” in Fig. 2A) shows that expression extends throughout almost the entire neural plate (Fig. 2a). By 18 hpf, *fzd3a* expression has expanded to encompass the entire length of the neural tube, although expression remained strongest in the cranial regions (Fig. 2B). Optical sections again revealed that the *fzd3a* expression domain extends throughout the dorsal part of the neural tube (Fig. 2b). However, we found no evidence of *fzd3a* expression in migrating NCCs in trunk or tail at 30 hpf, although expression persists strongly throughout the CNS (Fig. 2C).

*fzd9b* expression is much weaker than that of the other two genes at 1-somite stage (Fig. 2D), although it includes most of the neural plate in anterior half of the embryos. A pair of expression domains outside of the neural plate (arrowheads in Fig. 2d) were considered not to include NC because they lie internal to the ectoderm. By 18 hpf, *fzd9b* expression is readily detected in a complex pattern within the fore- and midbrain; levels in the hindbrain and spinal cord are also much higher, and posteriorly the expression domain has extended to the tail (Fig. 2E). Interestingly, the dorsal part of the anterior CNS (marked by bracket) lacks *fzd9b* transcripts whilst the dorsal neural tube in the spinal cord clearly expresses *fzd9b* (Fig. 2e). As with *fzd3a*, we saw no evidence of *fzd9b* expression in migrating NCCs at 30 hpf, although expression is seen in sites including most of the CNS, the tail tip, and at low levels in muscle (Fig. 2F).

*fzd10* is expressed strongly in the neural plate at 1-somite stage, but expression is absent from the anteriormost region, likely corresponding to some or all of the forebrain, and is strongest in the anterior part (Fig. 2G) and lateral border (Fig. 2H) of the neural plate expression zone, consistent with possible involvement in NCC induction. In addition there is very strong expression in the tail bud, which also persists at 18 hpf (Fig. 2I). At later stages (18 hpf, 30 hpf), *fzd10* is strongly expressed and tightly restricted to the dorsal neural tube along the entire axis of the embryo (Fig. 2I, 2i, 2J). Once again, expression in migrating NCCs was not detected (Fig. 2J).

### Knockdown of *fzd3a, fzd9b* and *fzd10*

Given their expression patterns in the dorsal neural tube, *fzd3a, fzd9b* and *fzd10* were strong candidates for encoding the Wnt receptor(s) associated with melanocyte specification from the NCC. To test this hypothesis, we performed morpholino-mediated knockdown analysis of each gene; in addition, to allow for likely redundancy of function, we took advantage of the ease with which multiple gene knockdown can be performed using this technique in zebrafish, knocking down all three genes simultaneously. A splice site morpholino targeting *fzd3a* has already been reported [35], so we used this same morpholino for our studies. Our analysis of the cDNA and genomic data (Ensembl) for *fzd9b* and *fzd10* indicated that there were no intron-exon boundaries within the known coding regions of these two genes; consequently, we designed morpholinos against the predicted translation start (ATG) codon. Sequence information for these morpholinos is listed in Table 2. To examine the efficacy of *fzd3a-*MO we used reverse transcription-polymerase chain reaction (RT-PCR) to confirm the changed size of transcript due to mis-splicing as reported [35] (See Materials and Methods for detail). As shown in Fig. S3A, 10 ng of the *fzd3a-*MO was sufficient to substantially suppress normal splicing of *fzd3a* in our hands. To show that our *fzd9b-* and *fzd10*-MO can suppress translation of their target sequences in vivo, we designed fusion constructs containing target sites of these morpholinos and an *enhanced green fluorescent protein* (*egfp*) reporter gene (Schematic drawing in Fig. S3B). Co-injection of morpholinos and synthesized RNA encoding fusion constructs containing the target sequence demonstrated that even 1 ng of *fzd9b-* or *fzd10*-morpholino was sufficient to suppress EGFP fluorescence derived from 100 pg of fusion construct mRNA (EGFP-positive embryos, 0/31 for *fzd9b-*MO injected; 0/23 for *fzd10*-MO injected) (See Fig. S3D, F, S3D', F'). In contrast, EGFP protein was detected in a significant proportion of embryos injected with even 5 ng of random control morpholino in both cases (EGFP-positive embryos, 73/93 for *fzd9b-*MO experiment; 23/28 for *fzd10*-MO experiment) (See Fig. S3C, E, 3C', E'). Although these data indicate the efficacy of our morpholinos against their chosen targets, morpholino knockdown of each of *fzd3a, fzd9b* or *fzd10* alone showed no noticeable reduction of melanocytes (Figure S4 and Table S1).

**Figure 3:**
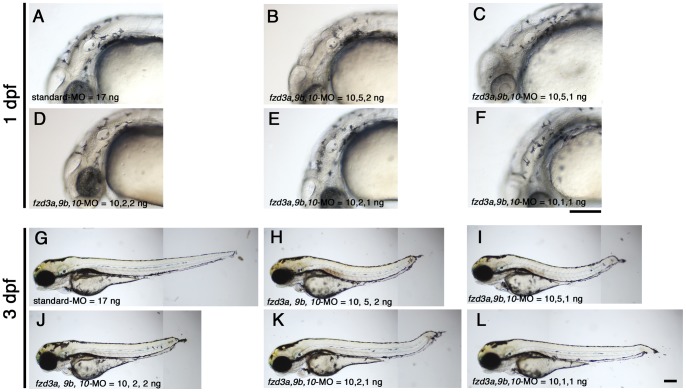
Combinatorial injections of *fzd3a, fzd9b* and *fzd10–*MO do not affect melanisation. A–F) 1 dpf embryos. Typical melanisation patterns of embryos showing “elongated axis+no/slight necrosis” phenotype in Table 3 are presented here for each injection. G–L) 3 dpf embryos. All are left side views with dorsal oriented to the top. A, G) Control standard-MO, 17 ng/embryo. B, H) *fzd3a, fzd9b, fzd10*-MO  = 10, 5, 2, ng/embryos, respectively. C, I) *fzd3a, fzd9b, fzd10*-MO  = 10, 5, 1, ng/embryos, respectively. D, J) *fzd3a, fzd9b, fzd10*-MO  =  10, 2, 2, ng/embryos, respectively. E, K) *fzd3a, fzd9b, fzd10*-MO  = 10, 2, 1, ng/embryos, respectively. F, L) *fzd3a, fzd9b, fzd10*-MO  =  10, 1, 1, ng/embryos, respectively. Scale bars: 200 µm.

We tested for functional redundancy of these three genes in this process by co-injecting all three morpholinos simultaneously to achieve multiple gene knockdown. In initial experiments, we experimented with differing amounts of MOs for each gene. Injected embryos were examined at 1 day post-fertilization (dpf) (Fig. 3A–F and Table 3) and 3 dpf (Fig. 3G–3L), noting the presence of and pattern of melanocytes, as well as morphological abnormalities. Since we knew that 10 ng/embryo of *fzd3a-*MO efficiently inhibited *fzd3a* splicing, we fixed the amount of *fzd3a-*MO to 10 ng/embryo, and varied the amounts of the others. The combined injection of 10 ng of *fzd3a*-MO and 5 ng of *fzd10*-MO resulted in substantial necrosis, so we used only doses of 1 or 2 ng/embryo of *fzd10*-MO in this experiment. Although combinatorial injection of these MOs caused a percentage of embryos to show severe necrosis and in some cases a severely truncated or bended body axis, in all cases at least c. 50% of injected embryos showed an elongated body axis and no or only minimal necrosis restricted to the brain. Surprisingly, we saw no reduction in melanocytes in those embryos with normal morphology, even in the embryos injected with the highest amounts of morpholino (i.e. *fzd3a, fzd9b, fzd10*-MOs at 10, 5, 2 ng/embryo, respectively; compare Fig. 3B, 3H with Fig. 3A, 3G.). We found less pigmented embryos in some cases, but all of these showed very severe morphological defects (Table 3; data not shown). Therefore, we discounted these pigmentation defects as secondary defects of disruption of early development. The embryos injected with lower doses of morpholinos also showed no reduction of pigmentation (Fig. 3C–F, 3I–L). The significant developmental abnormalities seen in the doses used here precluded the further increase in MO doses.

**Table 3:** Melanisation occurs in embryos injected with *fzd3a, 9b, 10*-MO at 1 dpf.

Type/Amount of MO	Elongated axis+ no/slight necrosis	Elongated axis+ severe necrosis	Shortened axis+ severe necrosis	Dead	Total
Standard-MO 17 ng/embryo	45 (45)	0 (0)	0 (0)	1	46
*fzd3a, 9b, 10*-MO = 10, 5, 2 ng/embryo	34 (34)	21 (21)	12 (5)	1	68
= 10, 5, 1 ng/embryo	23 (23)	24 (24)	0 (0)	1	48
= 10, 2, 2 ng/embryo	65 (65)	19 (19)	3 (1)	0	87
= 10, 2, 1 ng/embryo	32 (32)	9 (7)	7 (1)	3	51
= 10, 1, 1 ng/embryo	24 (24)	11 (8)	2 (1)	0	37

Notes.

Numbers in parenthesis are of significantly melanised embryos. We did not inject 5 ng/embryo or more of *fzd10* MO with other morpholinos because we found combinatorial injection of 10 ng of *fzd3a-MO* and 5 ng of *fzd10*-MO caused severe necrosis (data not shown). Here, “severe” necrosis means we found necrotic cell death everywhere in the body, whilst “slight” means necrosis was observed mainly in the head. Most embryos show elongated body axis, and those are categorised into “elongated axis”; others, categorised as “shortened axis”, lack tail and sometimes have severely bent or reduced trunk.

One possible explanation for our failure to see the expected melanocyte specification phenotype was that the MOs failed to inhibit Fzd function sufficiently. Our efficacy controls (Fig. S3) argue strongly against this caveat, but we also performed a direct test of the MOs' efficacy in reducing canonical Wnt signaling in vivo at the expected time of Wnt signaling. We injected embryos generated by intercrossing hemizygous TopdGFP transgenic fish with *fzd3a, fzd9b, fzd10*-MOs, and examined *gfp* expression by in situ hybridization analysis. This strain contains a transgene combining multiple Lef1-binding sites followed by a destabilized *gfp* cDNA, and has been shown to be a good in vivo reporter of Wnt activity [43]. In these crosses, we expected embryos to be homozygotes, hemizygotes and non-transgenic; consistent with this, we were able to categorize the signal intensity of GFP expression into three categories, strong, weak and no expression. Figure S5 shows typical examples of embryos showing “strong” (Fig. S5A) and “weak” (Fig. S5B, C) expression, and Table S2 describes the distribution of embryos categorized into these three categories. As shown in this table, the number of embryos classified as “strong” expression is reduced by the co-injection of *fzd3a, fzd9b* and *fzd10* MOs (2 out of 37) compared with the controls (11 out of 37). Statistical testing confirmed that the proportion of the three GFP expression patterns deviated from the expected Mendelian ratio (1∶2∶1), and the one observed in *fzd-3, fzd9b, fzd10*- morphant was significantly different from the prediction (Chi-squared, p = 0.022), whilst the one for control (standard morpholino-injected) was not (p = 0.868). We conclude that co-injection of the three *fzd* MOs substantially reduces Wnt signaling in vivo.

**Figure 4:**
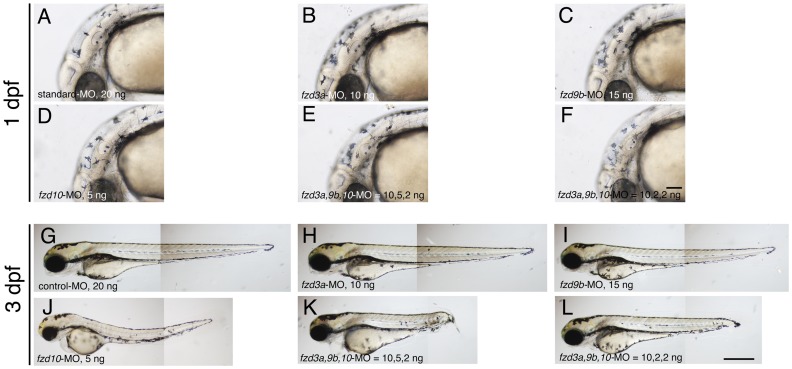
Morpholino-mediated knockdown of *fzd3a, fzd9b* and *fzd10* in the sensitized background. A–F) 1 dpf embryos. Melanisation patterns of typical embryos showing “elongated axis + no/slight necrosis” are presented here (see Table 4 for quantitation). G–L) 3 dpf embryos. All are left side views with dorsal oriented to the top. A, G) standard Control-MO, 20 ng/embryo. B, H) *fzd3a-MO* injected. 10 ng/embryo. C, I) *fzd9b-*MO injected. 15 ng/embryo. D, J) *fzd10*-MO injected. 5 ng/embryo. E, K) *fzd3a, fzd9b, fzd10*-MO  = 10, 5, 2, ng/embryo, respectively. F, L) *fzd3a, fzd9b, fzd10*-MO  = 10, 2, 2, ng/embryo, respectively. Scale bars: (A–F) 100 µm, (G–L) 500 µm.

Whilst our data clearly indicates that we achieved very substantial knockdown of Wnt signaling, we considered the possibility that nevertheless the remaining residual activity (See Fig. S5C) was sufficient to drive normal melanocyte specification. Current experimental data indicates that melanocyte specification depends upon a combination of Wnt signaling [44] and Sox10 activity [34], [38]. We thus reasoned that co-injection of the *fzd* MOs into *sox10* mutant heterozygotes might provide a sensitized background in which the roles for these *fzd* genes in melanocyte specification would be revealed. We crossed identified *sox10* mutant heterozygotes with wild types, and injected the resultant embryos (expected to be 50% *sox10* heterozygotes; 50% wild-types) with *fzd3a, fzd9b, fzd10*-MOs at various concentrations, alone or in combination. We quantitated the numbers of embryos showing reduced melanisation, expecting at most 50% of the injected embryos show reduced melanisation. Surprisingly, we again saw that almost all of the injected embryos showed normal melanisation at 1 dpf (Fig. 4B–F) and 3 dpf (Fig. 4H–L)(See also Table 4).

**Table 4:** Melanisation occurs in *sox10* mutant heterozygous embryos injected with *fzd3a, 9b, 10*-MO at 1 dpf.

Amount of MO	Elongated axis+ no/slight necrosis	Elongated axis+ severe necrosis	Shortened axis+ severe necrosis	Dead	Total
standard-MO 20 ng	37 (37)	0 (0)	0 (0)	0	37
*fzd3a*-MO 10 ng	48 (48)	0 (0)	0 (0)	1	48
*fzd9b*-MO 15 ng	49 (49)	0 (0)	0 (0)	1	50
*fzd10*-MO 5 ng	30 (30)	14 (13)	0 (0)	1	45
*fzd3a, 9b, 10*-MO = 10, 5, 2 ng	22 (22)	19 (17)	1 (0)	0	42
= 10, 2, 2 ng	22 (22)	15 (15)	0 (0)	3	40

Notes.

50% of embryos examined for each category should be *sox10* mutant heterozygotes. Numbers in parenthesis are of significantly melanised embryos. See Table 3 for definition of categories.

Taken together, we conclude that our data argue against a major role for *fzd3a, fzd9b* or *fzd10* in melanocyte specification (or subsequent differentiation). It seems likely that ubiquitously expressed *fzd* genes (e.g. *fdz6, fzd7a, fzd9a*) and/or low level expression of other *fzd* genes, or other types of receptors, transduce sufficient Wnt signaling for melanocyte development (see Discussion).

### Examining the role of *fzd* genes in NCC induction

**Figure 5:**
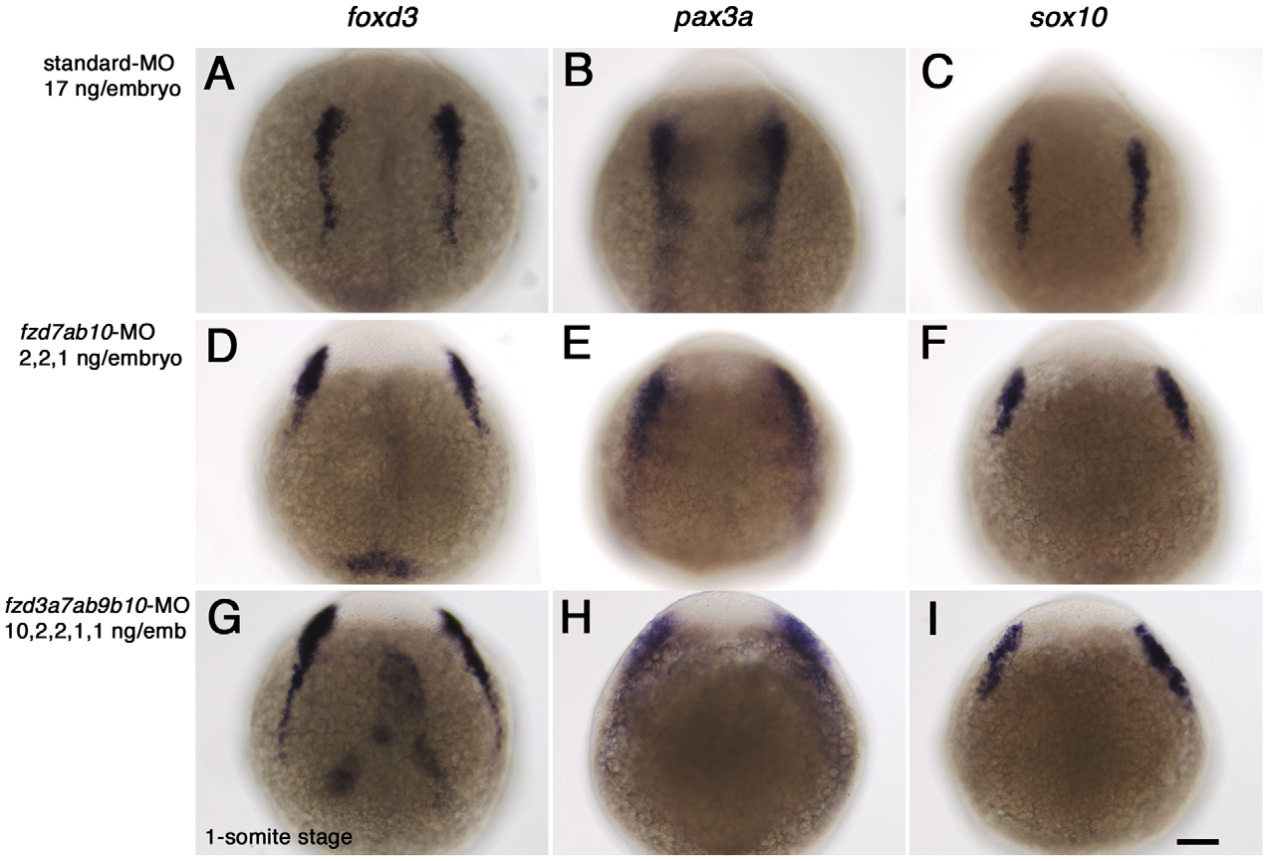
*fzd* gene knockdown has no effect on NCC induction, but causes CE defects. Embryos were injected with standard control-MO alone (A, B, C), or a mixture of *fzd7a, fzd7b*, and *fzd10*-M (D, E, F) or *fzd3a, fzd7a, fzd7b, fzd9b* and *fzd10*-MO (G, H, I), respectively. The amounts of each morpholino were as indicated. Embryos were examined by in situ hybridization analysis at 1-somite stage with three early NCC markers, *foxd3* (A, D, G), *pax3a* (B, E, H) and *sox10* (C, F, I). All are dorsal views with anterior oriented to the top. Scale bar: 100 µm.

In our analysis of the expression patterns of *fzd* genes, only *fzd7b* and *fzd10* gene showed expression in the lateral border of neural keel at 1-somite stage (Fig. 1Ga' and Fig. 2H). We thus hypothesized that these genes might mediate Wnt signaling during NC induction, and tested this idea by using morpholino-mediated knockdown. In addition, given the possibility of functional redundancy between *fzd7a* and *fzd7b*, we knocked down both *fzd7a* and *fzd7b* genes simultaneously. Morpholinos against the two *fzd7* genes have already been reported [28], and some other morpholinos against these were also examined by the same group. So we chose morpholino sequences based on their observations [28] (V. Korzh, pers. comm.) (for sequences, see Table 1). As marker genes for early NCCs, we used *foxd3, pax3* and *sox10*. These three genes encode transcription factors of the winged helix, paired box containing and HMG group transcription factor families respectively, and all have been shown to be expressed in premigratory NCC and to be critical factors for development of neural crest derivatives [34], [38], [45], [46], [47]. Importantly, previous reports suggested that their expression was dependent upon Wnt signaling [26], [48]. Fig. 5 shows the expression pattern of these three genes in control MO-treated embryos at 1-somite stage (Fig. 5A–C). Simultaneous inactivation of *fzd7a*, *fzd7b* and *fzd10* has no noticeable effect on the intensity of marker gene expression. However, these experiments clearly showed that the lateral spacing between the bilateral marker domains was increased in *fzd* MO-treated embryos, suggesting that CE movements during gastrulation might be disrupted [49], [50].

We then considered the possibility that the lack of a NCC induction phenotype might be due to other *fzd* genes. In particular we noted that *fzd3a* and *fzd9b* are also expressed in the neural plate, though these two are not restricted to the border between neural plate and epidermis (Fig. 2A, 2a, 2D, 2d). Consequently, we examined the effect of co-injection of *fzd3a, fzd7a, fzd7b, fzd9b and fzd10*-MO on NCC marker expression at 1-somite stage (Fig. 5G–I), but again saw no defect in NCC marker expression pattern or intensity, although the wider spacing of the lateral marker domains was seen. Interestingly, subsequent careful examination of the expression pattern of *fzd10* in comparison with the early NCC marker *foxd3* by double in situ hybridization, showed that the *fzd10* expression domain does not in fact overlap *foxd3* at this stage (See Figure **S2**). From these observations, we conclude that knockdown of *fzd7a, fzd7b* and *fzd10* is insufficient to affect NCC induction (See Discussion), but might be important for CE movements.

### Co-operative involvement of *fzd7a* and *fzd7b* on CE movement and requirement for *fzd7a*, *fzd7b* and *fzd10* in mesoderm formation

**Figure 6:**
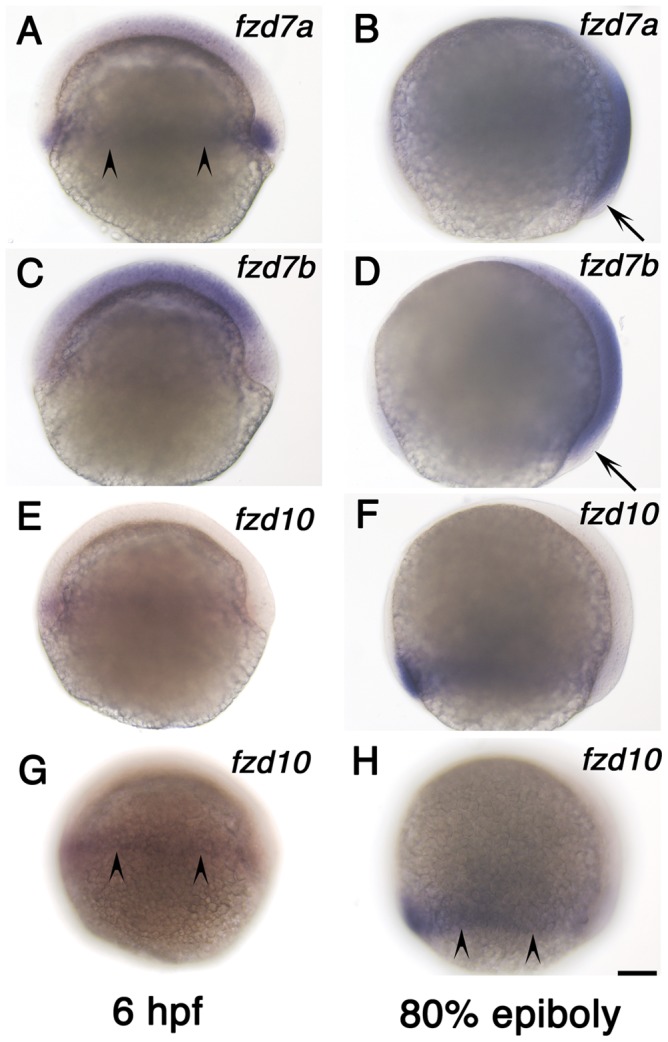
Expression of *fzd7a*, *fzd7b* and *fzd10* at gastrula stage. A, B) *fzd7a* expression. C, D) *fzd7b* expression. E–H) *fzd10* expression. Left column shows expression at 6 hpf and right column shows expression at 80% epiboly stage as indicated. All are left side views with anterior to the top. Embryos in panel E and G, and embryos in panel F and H are the same ones. We focused on medial plane along the animal-vegetal axis for embryos in panel A–F. Embryos in panel G and H alone were focused on lateral surface to show signal in marginal area. Arrowheads in panels A, G, H indicate the marginal expression, and arrows in B and D show the lack of expression (see text). Scale bar: 100 µm.

To investigate the requirement of these three *fzd* genes in CE, we first examined their expression at early gastrula stages when CE movements occur [51], [52]. All three *fzd* genes were expressed in gastrula stages, but each had a unique pattern (Fig. 6). At 6 hpf, *fzd7a* and *fzd10* are expressed in the blastoderm margin (arrowheads in Fig. 6A for *fzd7a* and in Fig. 6G for *fzd10*), whilst *fzd7b* expression was throughout the blastoderm but excluded from the marginal area (Fig. 6C). Like *fzd7b* (Fig. 6C), *fzd10* expression is reduced or lacking from the embryonic shield (Fig. 6E, G). At the 80% epiboly stage, the expression domains of *fzd7a* and *fzd7b* expand to become widespread throughout the entire embryo, except for the marginal region of the dorsal blastoderm (arrows in Fig. 6B, D). In contrast, *fzd10* expression remains restricted to the ventro-lateral marginal zone of blastoderm (arrowheads in Fig. 6H). These in situ analyses clearly demonstrate expression patterns of these three *fzd* genes that are fully consistent with their possible involvement in CE movement.

**Figure 7:**
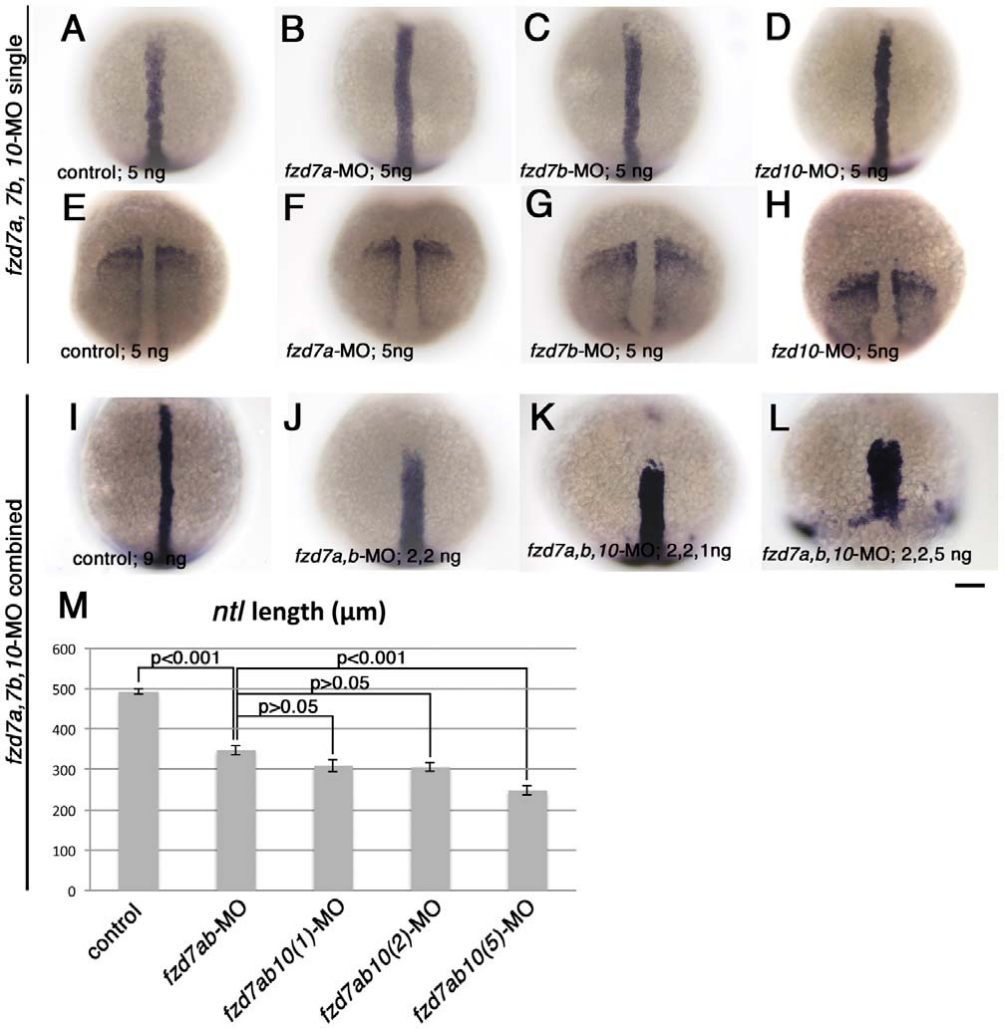
Combinatorial injection of *fzd7a* and *fzd7b-*MO causes dramatic defects in mesodermal CE. Embryos were processed to show expression of notochord marker *ntl* (A–D, I–L), and somatic and pre-somitic mesoderm marker, *papc* (E–H). All embryos are 1-somite stage, and oriented with their animal pole toward top. Embryo in panel L was fixed at the time when control embryos reached 1-somite stage. All are dorsal views. Amount of each morpholinos injected are indicated in bottom left corner of each panel. Scale bar: 100 µm. M) Quantitation of length of dorsal midline *ntl* expression domain. The amount of morpholino in each case were as follows. Control-MO, 9 ng; *fzd7a, fzd7b*-MOs, 2 ng each; *fzd7a, fzd7b, fzd10*-MOs, 2, 2, 1 ng, respectively; *fzd7a, fzd7b, fzd10*-MOs, 2, 2, 2 ng, respectively; *fzd7a, fzd7b*, *fzd10*-MOs, 2, 2, 5 ng, respectively. P values estimated by Tukey's multiple comparison test are indicated for each pair. Error bars are s.e.m. for each group.

The expression patterns described in Fig. 6 suggest that, in addition to the CE defects we have observed in the ectoderm (Fig. 5), we might expect CE defects in the mesoderm too. To test this, we examined the standard markers used to assess mesodermal CE movements, *no tail* (*ntl*) [53] and *paraxial protocadherin* (*papc*) [54]. *ntl* is expressed in the blastoderm margin and the presumptive notochord from early gastrula to 1-somite stage, whilst *papc* is strongly expressed in paraxial mesoderm and excluded from the dorsal midline. Previous studies have shown that the *ntl* notochord expression domain becomes shortened along the antero-posterior axis and widened mediolaterally when CE is inhibited [55], [56]. Conversely, the spacing between the two *papc* expression domains becomes wider and the expression domains shorter where CE is disrupted [49]. At first, we knocked down *fzd7a, fzd7b* and *fzd10* separately by injecting with 5 ng of morpholino for each. Figs. 7A–7D and 7E–7H show the expression pattern of *ntl* (7A–7D) and *papc* (7E–7H) in each case at 1-somite stage. As shown here, *fzd7a* and *fzd7b*-MO have no significant effect on the length and width of these expression patterns (compare panel 7A or 7E with 7B, 7C or 7F, 7G), though *papc* expression seems slightly shortened in *fzd10*-morphants (Fig. 7H).

Since we expected functional redundancy between *fzd7a* and *fzd7b*, we next tested experimentally the effects of double knockdown of *fzd7* function, and also co-injection with a series of doses of *fzd10*-MO with the *fzd7a, fzd7b* MOs in order to assess the co-operative action of these three genes. We injected embryos with standard-MO as control (Fig. 7I), or *fzd7a-*MO and *fzd7b-*MO together (Fig. 7J), or *fzd7a-*MO, *fzd7b-*MO and *fzd10-*MO together (Fig. 7K, 7L) and examined the expression of *ntl* in embryos fixed at 1-somite stage. The combined injection of only 2 ng each of *fzd7* morpholinos resulted in dramatic shortening of the *ntl* domain (compare Fig. 7I and 7J), consistent with a CE defect. To quantify this phenotype, we measured the length of the notochord domain of *ntl* in these embryos (Fig. 7M). Statistical analysis by one-way ANOVA and further Tukey's multiple comparison analysis confirms the statistically significant reduction of notochord length in the *fzd7a, fzd7b* double morphants compared with control (p<0.001). These data strongly suggests that *fzd7a* and *fzd7b* act in a functionally redundant manner to mediate Wnt signals important for CE movement. Next, we assessed whether *fzd10* might act in addition to the two *fzd7* genes to contribute towards this role in CE movement. When compared with the control embryo (Fig. 7I), combined injection of *fzd7a, fzd7b* and *fzd10*-MOs resulted in a severely truncated and broadened *ntl* expression domain, although this was only seen with 5 ng of *fzd10*-MO (Fig. 7L, 7M) and not with 1 or 2 ng doses (compare Fig. 7J and 7K; quantitation in Fig. 7M). Further, we also observed an effect on epiboly, with 5 ng of *fzd10*-MO causing a delay in progression of epiboly, and reduction in *ntl* expression in the marginal zone (compare Fig. 7I and 7L). From these observations, we conclude that *fzd7a* and *fzd7b* are the primary Wnt receptors driving proper CE movement during gastrulation, and that *fzd10* does not have a major role. However, reduction of *ntl* expression in embryos injected with higher amounts of *fzd10*-MO as well as both *fzd7a* and *fzd7b*-MOs suggests that all act together to regulate epiboly movements and early mesoderm differentiation.

## Discussion

In this study, we have comprehensively surveyed the expression patterns of the *fzd* genes in zebrafish development up to 30 hpf. In doing so, we identify discrete and diverse expression domains for most of these genes, although there is much overlap at specific times and in specific organs, and three genes show apparently ubiquitous expression at at least one stage of the three we have focused on. These data are consistent with the diverse and repeated uses of Wnt signaling in development, and the challenge now is to relate individual *fzd* gene expression patterns to specific roles of Wnt signaling. In an attempt to begin to address this challenge, we focused initially on two well-characterized processes known to be dependent upon Wnt signaling, NCC induction and melanocyte specification from the NC.

We identified *fzd3a, fzd9b* and *fzd10* as being expressed in the dorsal neural tube, where melanocyte specification likely occurs. Unexpectedly, we were unable to identify conditions of single or combinatorial morpholino-mediated knockdown of these Fzds that resulted in phenotypes consistent with an inhibition of melanocyte specification. Likewise, we identified *fzd7b* and *fzd10* as being expressed in the neuro-ectodermal border at 1-somite stage, and tested these for a role in NCC induction by morpholino-mediated knockdown. Again, unexpectedly we were unable to identify a clear role for either of these genes in NC induction. However, these experiments identified a clear phenotype in CE movement, suggesting that *fzd7a, fzd7b* and *fzd10* act in a partial redundant manner to mediate this aspect of Wnt signaling.

### Are *fzd* genes required for NCC induction and melanocyte specification?

Our experiments using morpholino combinations to knockdown up to five (*fzd3a, fzd7a, fzd7b, fzd9b,* and *fzd10*) or up to three (*fzd3a, fzd9b* and *fzd10*) Fzd proteins failed to result in detectable reduction of either NCC or melanocyte formation, respectively. Nevertheless, we have evidence that our morpholinos were generating substantial knockdown of the targets. Firstly, the absence of normal length transcripts for *fzd3a* after injection of *fzd3a-*MO suggests this morpholino was successfully suppressing normal splicing. Secondly we observed a CE defect and reduction of mesodermal markers when *fzd7a, fzd7b, fzd10*-MO were injected into embryos. Thirdly, we showed that our *fzd9b-*MO could bind to the target sequence and suppressed the translation of an *egfp* reporter gene at doses of only 1 ng per embryo, which is much less than the amount injected for analysis. Fourthly, reduction of GFP reporter expression in TopdGFP transgenic analysis injected with *fzd3a, fzd9b, fzd10*-MO indicated that we were achieving substantial suppression of the canonical Wnt pathway. Finally, in *fzd3a, fzd9b, fzd10*-morphants, we found frequent tail fin defects (see Fig. 3, 4), resembling those seen after disruption of Wnt signaling by double knockdown of *lef1* and *tcf7* [57]. Taken together, these observations strongly suggest that our morpholinos were effective, and thus that the lack melanocyte and NCC induction phenotypes provides strong evidence indicating that these *fzd* genes are unlikely to play a major role in these processes.

Given the robust evidence for Wnt signaling involvement in NC induction and melanocyte specification, this forces the question of whether other *fzd* genes are involved in these developmental aspects. Mammals have 10 *fzd* genes and since an extra whole-genome duplication event occurred in the teleost lineage compared with that of mammals, it is conceivable that zebrafish may have 20 (or more) *fzd* genes in total. Our comprehensive BLAST search used the previous (Zv8) version of the zebrafish genome database; repeating our search using the latest version (Zv9) did not find any new *fzd* genes. However, we cannot rule out there being *fzd* genes still to be identified. In particular, other paralogues may be identified as the genome sequence becomes refined. It is known that duplicated genes may have (partially) divergent expression patterns in zebrafish, so that only one of the duplicated genes retains specific ancestral functions [58]. Any novel *fzd* genes should be directly assessed for candidacy as receptors mediating NCC induction and melanocyte specification, by expression and functional analysis.

Our assessment criteria relied on specific expression at specific locations at specific times; it is conceivable that we did not detect the crucial *fzd* genes, for example, due to lower levels of expression in these locations. We did not perform comprehensive assessment of Fzd1, Fzd2, Fzd4, Fzd5, Fzd6 and Fzd8 for roles in melanocyte development or NCC induction. Where the expression patterns of other vertebrate *fzd* genes correspond to the time and place of NC induction or melanocyte specification, this might indicate which other *fzd* genes should be assessed more carefully. In mice, for example, *Sox10* is reported to be expressed in dorsal neural tube from E8.5 to E10.5 [59] so that *fzd* genes expressed during these stages will be potential receptors required for melanocyte specification. The data from mouse does not currently provide evidence that any of *Fzd1, Fzd2, Fzd4, Fzd5, Fzd6* or *Fzd8* show appropriate expression patterns [60], although studies to date have not focused on NC development. In the case of NC induction, *fzds* expressed in the neuro-ectodermal border around E8 stage might be strong candidates, since this is when expression of *Msx* and *Zic*, two early markers of the NC, are seen [61], [62], [63], [64], [65]. However, again the mice data do not currently implicate any of the *fzd* genes which were not knocked down here in NC induction, since the described patterns encompass mesodermal lineages (*Fzd1*, *Fzd6*, *Fzd8*) [60], future ventral neural tube (*Fzd1*) [60] or telencephalon (*Fzd5*) [9], [60]. Fzd2 alone has been reported to be expressed in the entire neural plate (including NCC contributing to the cardiac outflow tract [66]). We found no sign of *fzd2* expression in the zebrafish neural tube at 1-somite nor 18 hpf stage; thus, a duplicated *fzd2* gene might be a strong candidate gene for zebrafish melanocyte specification. Our assumption that the key receptors would be *specifically* expressed in the lateral neural plate or in the early NCCs may also be mistaken; we dismissed *fzd* genes showing ubiquitous expression from further study. Thus, *fzd6, fzd7a* and *fzd9a* might be worthy of further assessment in this context, although it seems likely that if they are crucial they are likely to be at least partially redundant amongst themselves.

Our data at least hint at the possibility that Wnt signals mediating NCC induction and melanocyte specification might depend primarily on receptors belonging to families other than the Frizzled family. As mentioned before, LRP5, 6 also function as Wnt receptors, although they appear to function as co-receptors for Fzd receptors, rather than functioning as an independent receptor [2], [67]. Other studies have recently revealed two other Wnt receptors, Derailed/Ryk and Ror, which can transduce Wnt signal in a Fzd-independent manner [2], [68]. The Derailed/Ryk family transmembrane proteins are atypical receptor type tyrosine kinases, and have been studied so far in *D. melanogaster* (Derailed), *C. elegans* (Lin-18) and vertebrates (Ryk). Phenotypic analysis in *Drosophila* and *C. elegans* [69], [70] and biochemical analyses [70], [71] provided evidence of genetic and physical interaction between Wnt and Ryk protein in a Fzd-independent manner. Functional analyses have revealed that Derailed/Ryk were involved in axon guidance in *Drosophila* [70] and mouse [71], in vulval formation in *C. elegans* [69], and in CE movement in *Xenopus* and zebrafish embryo [72], [73]. However, the evidence published to date suggests no compelling argument for the requirement of Ryk protein in the development of NCCs and their derivatives. Embryonic expression patterns of *ryk* genes in *Xenopus* and zebrafish show their strong enrichment in somite and central nervous system; expression is not enriched in dorsal neural tube at the times examined in this study, although *ryk* seems to be weakly and ubiquitously expressed in zebrafish at 19 hpf [73], [74]. Another Wnt receptor, Ror, is also a transmembrane tyrosine kinase, which contains Fzd-like cysteine-rich domain in the extracellular domain. Two structurally related proteins, Ror1 and Ror2, have been identified so far, and recent analyses have demonstrated evidence of involvement of Ror family kinases in Wnt signaling pathways, such as the physical interaction of Ror2 protein with Wnt5a [75], consistent with the similar phenotypes of *Ror2* and *Wnt5a* deficient mice [76], and genetic interaction between *cam-1* (*C.elegans* homolog of *Ror* ) and Wnt family gene *egl-20* [77]. Functional analysis further revealed that Ror mediated the changes in cell polarity and cell migration in CE movement that result from Wnt5a- and Wnt11- triggered non-canonical Wnt signaling [76], [78]. However, again, these studies provide no evidence for the involvement of Ror in NCC development. Rather, studies using cultured cells demonstrate Wnt5a-Ror2 signaling actually inhibits the canonical Wnt pathway, whilst Wnt5a can also activate the canonical pathway when the appropriate receptor (Fzd4) is available [75]. Taken together, we conclude that it is unlikely that zebrafish homologs of *Ror* genes mediate Wnt signaling in NC induction or melanocyte specification, although their expression patterns have not been reported yet. In zebrafish, MuSK (muscle-specific receptor kinase), a related protein to Ror [79], is mutated in *unplugged* mutants [80]; whilst it plays a role in controlling segmental migration of trunk NCCs, there is no report of defective NC induction or melanocyte specification in these mutants [81], [82].

In summary, knockdown of the subset of *fzd* genes on which we focused here is apparently insufficient to effect the reduction of melanocytes and NCCs, although the morpholinos themselves seem to be effective. We propose that further exploration, focused on ubiquitously expressed *fzds* or *fzd* gene expression at low levels, is now required to identify the crucial *fzd* receptor(s) mediating melanocyte development and NCC induction. Completion of the zebrafish genome sequencing project will clarify whether further *fzd* genes remain still to be considered in this context.

### Other roles of *fzd* genes

In addition to illuminating the role of *fzd* genes in CE movements, NC induction and melanocyte specification, our comprehensive analysis of *fzd* gene expression during somitogenesis and prim stages suggests further roles for specific genes. Here, we highlight specific aspects of the expression patterns of some *fzds*. *fzd4* shows very localised expression in the region of the otic and lateral line placodes (Fig. 1Da, Da', Da''). Cranial sensory placodes are, like NCCs, formed at the boundary of the neural plate and prospective epidermis. Development of the placodes is thought to start with induction of the preplacodal region, a ground state for all cranial sensory placodes. Subsequently, the preplacodal domain becomes subdivided into individual placodes, such as olfactory, lens, otic and lateral line placodes, by specific inducing signals [83]. Several lines of evidence suggested the involvement of Wnt signaling in this subdivision process to specify otic placode [84].

In contrast to otic development, the role of Wnts in the initial development of the posterior lateral line placode has been little studied in zebrafish, whilst neuromast formation in later stages has been intensively studied [85]. The *fzd4* expression pattern demonstrated here suggests the involvement of Wnt signaling in the initial formation of the lateral line placode. Intriguingly, *fzd4* seems to be expressed in progenitors for both the future otic and lateral line placodes. It is not clear whether otic and lateral line placodes are each induced from a shared set of progenitor cells in the preplacodal region, but this is one possible interpretation of the *fzd4* expression patterns we have observed at 1-somite stage, well before the emergence of morphologically-distinct otic and lateral line placodes. Knockdown studies of *fzd4* will be of interest for a test of this hypothesis.

Other interesting expression domains revealed here are those of *fzd5* and *fzd8a* in the ventral ( = subpalial) telencephalon. The nascent telencephalon is known first to be divided into dorsal and ventral parts, which then form the palium and subpalium, respectively [86]. This dorso-ventral patterning is followed by further development of different types of neurons along that axis [87]. Wnt/BMP (bone morphogenetic protein) and Hedgehog/FGF (fibroblast growth factor) signals are thought to play major roles in patterning of the dorsal and ventral regions respectively [86]. Our data showing expression of *fzd3b* and *fzd4* is consistent with this notion. On the other hand, *fzd5* (Fig. 1Ec) and *fzd8a* (Fig. 1Ic) are both expressed in the ventral part of the telencephalon. These observations clearly imply a role for Wnt signaling in the development of ventral telencephalon, too. Indeed, conditional knockout of*ß-Catenin* causes severe impairment of growth of the medial ganglionic eminence in the subpalium [88], so that this is at least one candidate role for *fzd5* and *fzd8a* in this region.

### Identification of new *fzds* required for CE movement

Here, we have demonstrated the co-operative requirement of *fzd7a* and *fzd7b* in CE movement. Since the important role of *fzd7* in CE movement has already been reported in *Xenopus* and mice [89], [90] and it is reasonable that duplicated genes share the function in a certain developmental event, this result reveals strong conservation of *fzd7* function in CE movements. In addition, we here identify *fzd10* as another Wnt receptor contributing to epiboly movement and mesodermal differentiation, working together with *fzd7a* and *fzd7b*. Although a recent publication reported the redundant function of *fzd2* and *fzd7* in CE movement in mice [90], co-operation of *fzd10* with *fzd7s* during gastrulation has not been reported so far. Furthermore, previous reports on zebrafish *fzd10* suggest this receptor activates the canonical Wnt pathway during early development [91], rather than the non-canonical Wnt pathway usually associated with cell movement. Therefore, co-operation of *fzd7a* and *fzd7b* with *fzd10* in regulating epiboly movements was unexpected. Nevertheless, analysis in human cancer cells demonstrates the promotion of cell motility through activation of the non-canonical pathway by FZD10 [92]. These observations support the idea that *fzd10* can transduce Wnt signaling through both canonical and non-canonical pathway dependent on the developmental context. As noted previously, switching between canonical and non-canonical Wnt signaling is, at least in certain cases, dependent upon the type of (co)receptor available [75]. Characterizing the co-receptors involved in these two examples may help with understanding the molecular nature of dual properties of *fzd10* in signal transduction.

In summary, our data define the roles of specific *fzd* genes in CE movements during gastrulation, and limit the candidates for Wnt receptors mediating NC induction and melanocyte specification. Furthermore, our expression data provides the basis for a targeted dissection of the role of Fzds in the embryonic development of numerous structures, some of which we have highlighted here.

## Supporting Information

Figure S1 **Probe regions in** ***fzd*** **cDNAs used in this study.** Solid black lines represent the entire cDNA reported (For accession numbers for these *fzds*, see Table 1). Figures on the right ends of each cDNA are total base pair numbers. White bars represent the cloned location to make probes. ATG and stop codon sites are shown here as predicted in registered information. Figures under each black line are base pair number from the first of the cDNAs.
(TIF)

Figure S2 ***fzd7b*** **expression domain overlaps future NCC marked by** ***foxd3*** **domain, whilst** ***fzd10*** **expression domain does not.** Here, *eya1* is used as placodal marker, and *foxd3* is used as NCC marker. Aa–Ac) Staining with *fzd7b* (purple) and *eya1* (red). Ba–Bc) *fzd10* (purple) and *eya1* (red). Ca–Cc) *fzd7b* (purple) and *foxd3* (red). Da–Dc) *fzd10* (purple) and *foxd3* (red). All images show dorsal views of right half of flat-mounted 1 -somite stage embryos. Anterior to the top. a, b, c for each data set represent bright field, dark field, and merged images, respectively. Note that there is a gap between *fzd10* expression domain and *eya1* expression domain (B), whilst *fzd7b* and *eya1* expression domains contact each other directly (A), suggesting *fzd7b* expression domain expands more laterally than *fzd10*. In C, *fzd7b* and *foxd3* are co-expressed (arrowhead). In contrast, *fzd10* and *foxd3* expression domains abut, but do not overlap (D). Scale bar: 100 µm.
(TIF)

Figure S3 **Testing efficacy of** ***fzd*** **gene morpholinos.** A) 10 ng of *fzd3a-MO* can substantially suppress the normal splicing of *fzd3a* mRNA. RT-PCR analysis was carried out as previously reported (See Materials and Methods). We injected 10 ng of morpholinos into wild-type embryos, and extracted total RNA at 24 hpf. Whilst control random oligo-injected (lane 2) and uninjected (lane 3) samples showed a clear *fzd3a* transcript band of the expected size (465 bp, arrowhead)., this band was barely detectable after 10 ng of *fzd3a-MO* injection due to the inhibition of correct splicing (lane 1). Primers against *β-actin* cDNA were used as a positive control for RNA extraction and RT-PCR (lanes 4, 5, 6.). B) Schematic drawing of fusion constructs to test ability of *fzd9b* and *fzd10* morpholinos to bind to the target sites and suppress translation in vivo. Target sites for *fzd9b-* and *fzd10*-MO (red) are fused to cDNA of *egfp* (green). Actual sequences of target site and the first 3 bps of *egfp* sequence are shown below the scheme. Original ATG for *egfp* gene is modified into ATC, which is next to GTG in green, in order to avoid translation from this. C–F, C'–F') Our *fzd9b-* and *fzd10*-MOs can efficiently suppress GFP fluorescence derived from injected fusion constructs. In all cases, 100 pg of mRNA of the respective fusion construct was injected, and embryos were observed at 10 hpf stage. GFP fluorescence was clearly detected in embryos injected with 5 ng of control random morpholinos (C, C', E, E'). In contrast, injection of even just 1 ng of the respective experimental morpholinos suppressed fluorescence completely (D, D', F, F'). C–F are bright field images of C'–F', respectively. Numbers in C'–F' are ratios of GFP-positive embryos out of surviving injected embryos. Scale bar: 500 µm.
(TIF)

Figure S4 **Phenotypes of embryos injected with** ***fzd3a, fzd9b*** **or** ***fzd10*** **-MO alone.** All are 32 hpf stages. Left side views with dorsal to the top. Name and the amount of each morpholino are shown in bottom left corner. A', B', C', D', E' are close up of the embryos shown in panels A, B, C, D, E, respectively. Scale bars: 100 µm.
(TIF)

Figure S5 ***gfp*** **expression in TopdGFP transgenic fish embryos injected with** ***fzd*** **-MOs.** A, B) Typical expression patterns of control embryos categorised as “strong” (A) and “weak” (B) group. C) Embryo co-injected with 10 ng of *fzd3a-MO*, 1 ng of *fzd9b-*MO and 1 ng of *fzd10*-MO. This embryo was categorized as “weak” expression. Left side view with anterior oriented to the top. Bud stage. See Table S2 for quantitation. Scale bar: 100 µm.
(TIF)

Table S1 **Injection of single** ***fzd-3a, 9b*** **or** ***10*** **-MOs has no effect on melanophore formation.**
(DOCX)

Table S2 **Combinatorial knock down of** ***fzd-3a, 9b, 10*** **causes reporter down-regulation in TopdGFP transgenic fish.**
(DOCX)
