# Supplementary figures and images for: A Systematic Survey of Expression and Function of Zebrafish frizzled Genes
Source: PLoS One. 2013 Jan 22;8(1):e54833. doi: 10.1371/journal.pone.0054833 (PMC3551900; doi:10.1371/journal.pone.0054833)

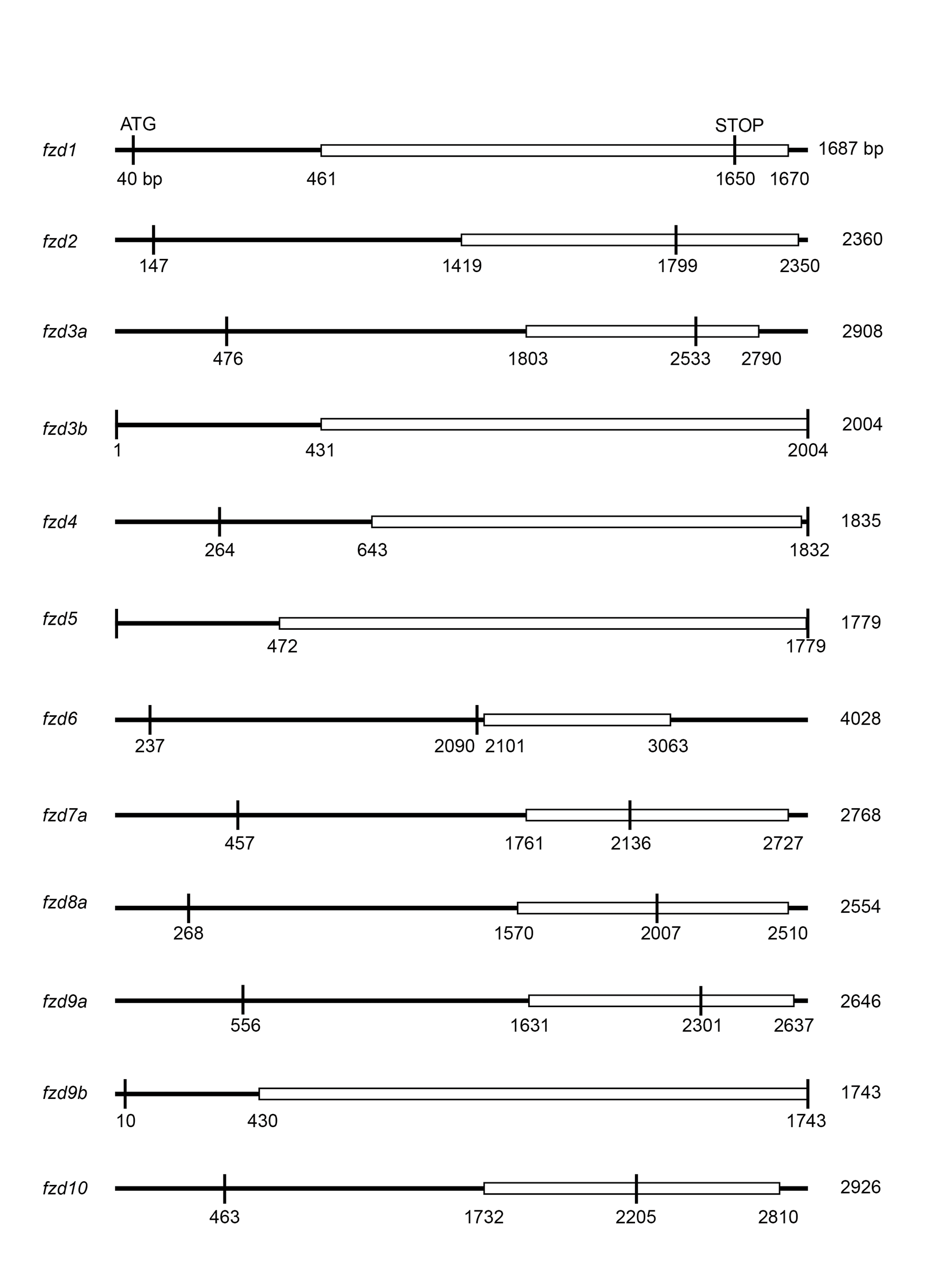

Supplement: Figure S1 — Probe regions in fzd cDNAs used in this study. Solid black lines represent the entire cDNA reported (For accession numbers for these fzds, see Table 1). Figures on the right ends of each cDNA are total base pair numbers. White bars represent the cloned location to make probes. ATG and stop codon sites are shown here as predicted in registered information. Figures under each black line are base pair number from the first of the cDNAs. (TIF) [file pone.0054833.s001.tif]

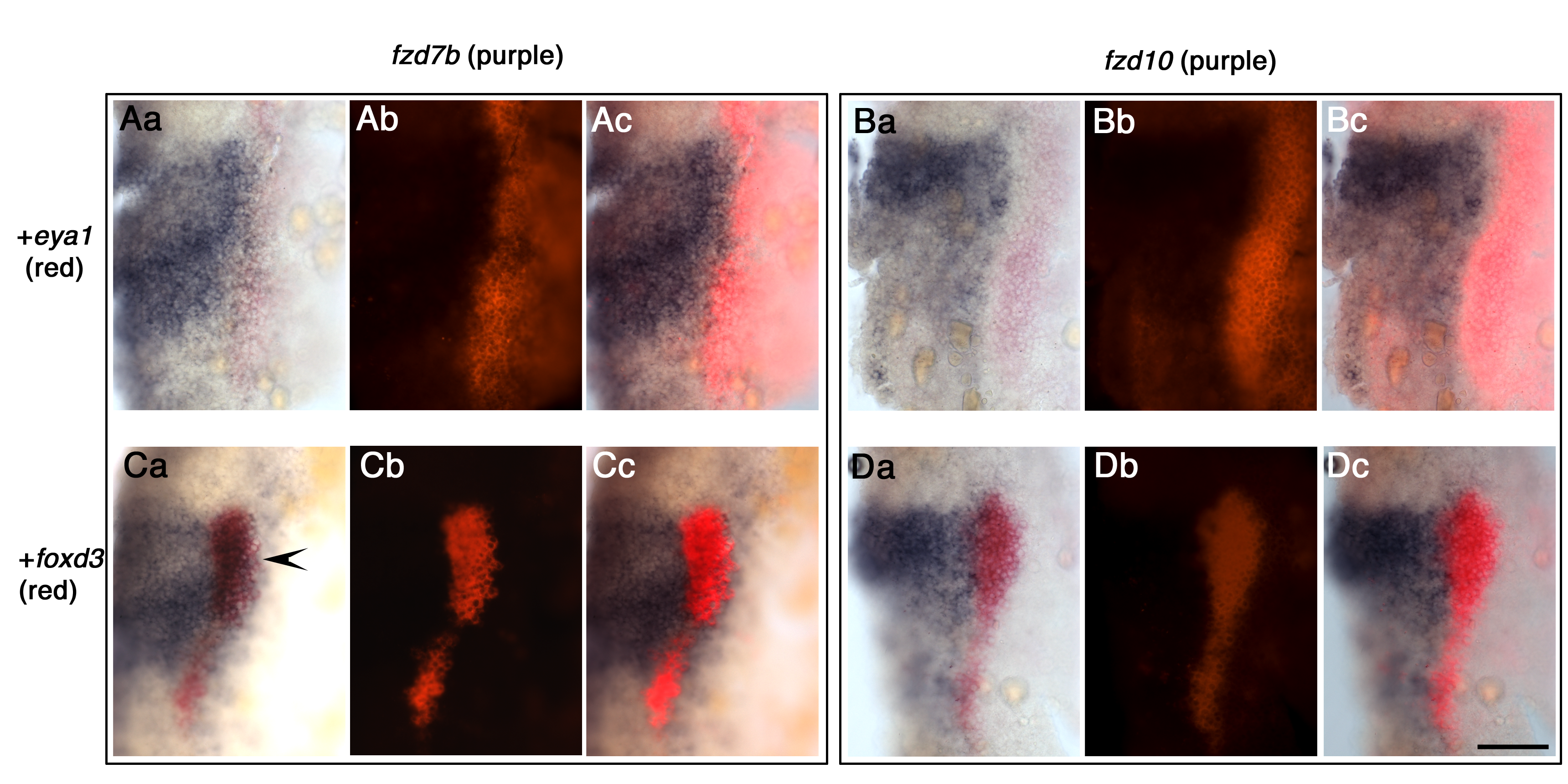

Supplement: Figure S2 — fzd7b expression domain overlaps future NCC marked by foxd3 domain, whilst fzd10 expression domain does not. Here, eya1 is used as placodal marker, and foxd3 is used as NCC marker. Aa–Ac) Staining with fzd7b (purple) and eya1 (red). Ba–Bc) fzd10 (purple) and eya1 (red). Ca–Cc) fzd7b (purple) and foxd3 (red). Da–Dc) fzd10 (purple) and foxd3 (red). All images show dorsal views of right half of flat-mounted 1 -somite stage embryos. Anterior to the top. a, b, c for each data set represent bright field, dark field, and merged images, respectively. Note that there is a gap between fzd10 expression domain and eya1 expression domain (B), whilst fzd7b and eya1 expression domains contact each other directly (A), suggesting fzd7b expression domain expands more laterally than fzd10. In C, fzd7b and foxd3 are co-expressed (arrowhead). In contrast, fzd10 and foxd3 expression domains abut, but do not overlap (D). Scale bar: 100 µm. (TIF) [file pone.0054833.s002.tif]

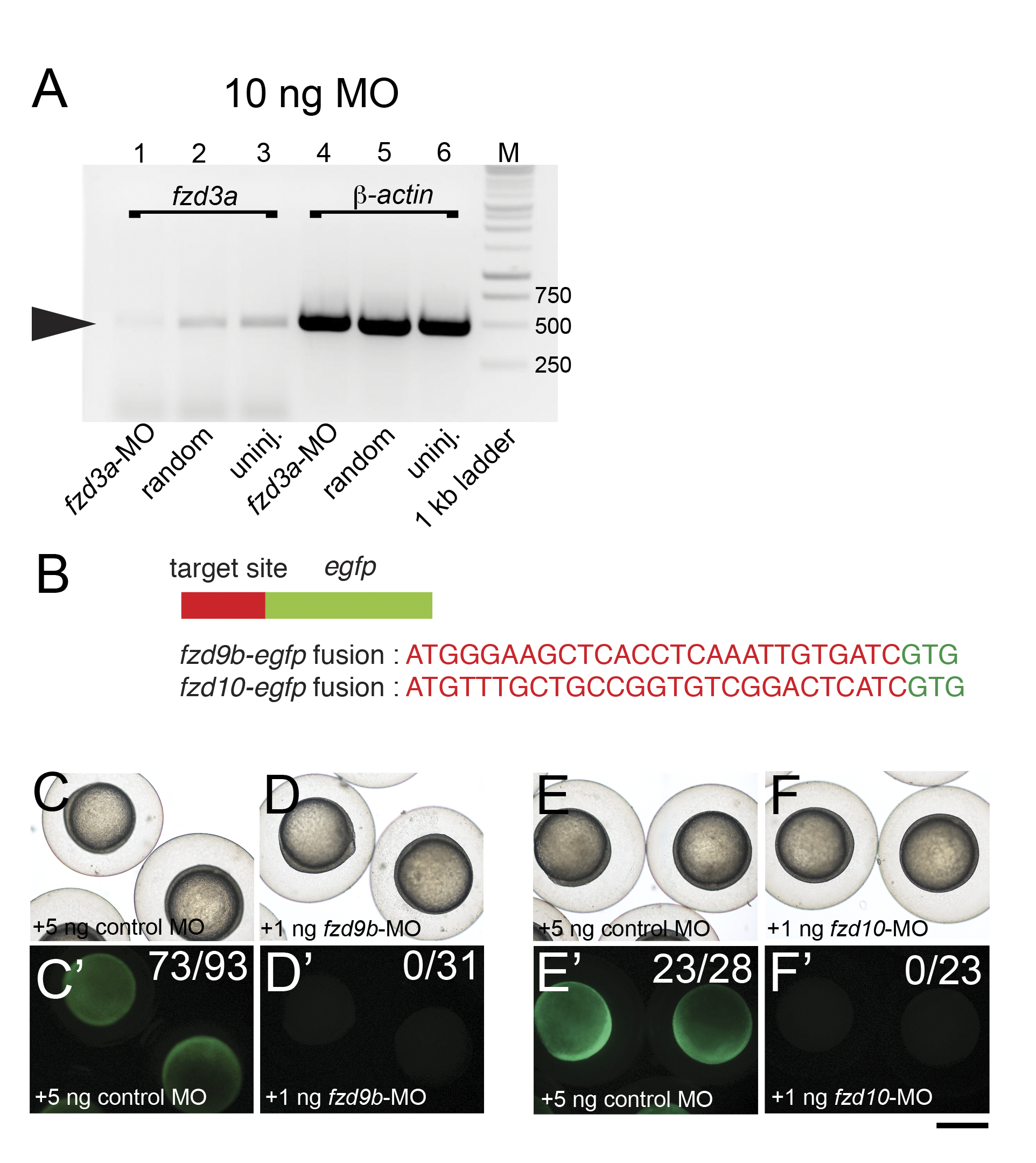

Supplement: Figure S3 — Testing efficacy of fzd gene morpholinos. A) 10 ng of fzd3a-MO can substantially suppress the normal splicing of fzd3a mRNA. RT-PCR analysis was carried out as previously reported (See Materials and Methods). We injected 10 ng of morpholinos into wild-type embryos, and extracted total RNA at 24 hpf. Whilst control random oligo-injected (lane 2) and uninjected (lane 3) samples showed a clear fzd3a transcript band of the expected size (465 bp, arrowhead)., this band was barely detectable after 10 ng of fzd3a-MO injection due to the inhibition of correct splicing (lane 1). Primers against β-actin cDNA were used as a positive control for RNA extraction and RT-PCR (lanes 4, 5, 6.). B) Schematic drawing of fusion constructs to test ability of fzd9b and fzd10 morpholinos to bind to the target sites and suppress translation in vivo. Target sites for fzd9b- and fzd10-MO (red) are fused to cDNA of egfp (green). Actual sequences of target site and the first 3 bps of egfp sequence are shown below the scheme. Original ATG for egfp gene is modified into ATC, which is next to GTG in green, in order to avoid translation from this. C–F, C'–F') Our fzd9b- and fzd10-MOs can efficiently suppress GFP fluorescence derived from injected fusion constructs. In all cases, 100 pg of mRNA of the respective fusion construct was injected, and embryos were observed at 10 hpf stage. GFP fluorescence was clearly detected in embryos injected with 5 ng of control random morpholinos (C, C', E, E'). In contrast, injection of even just 1 ng of the respective experimental morpholinos suppressed fluorescence completely (D, D', F, F'). C–F are bright field images of C'–F', respectively. Numbers in C'–F' are ratios of GFP-positive embryos out of surviving injected embryos. Scale bar: 500 µm. (TIF) [file pone.0054833.s003.tif]

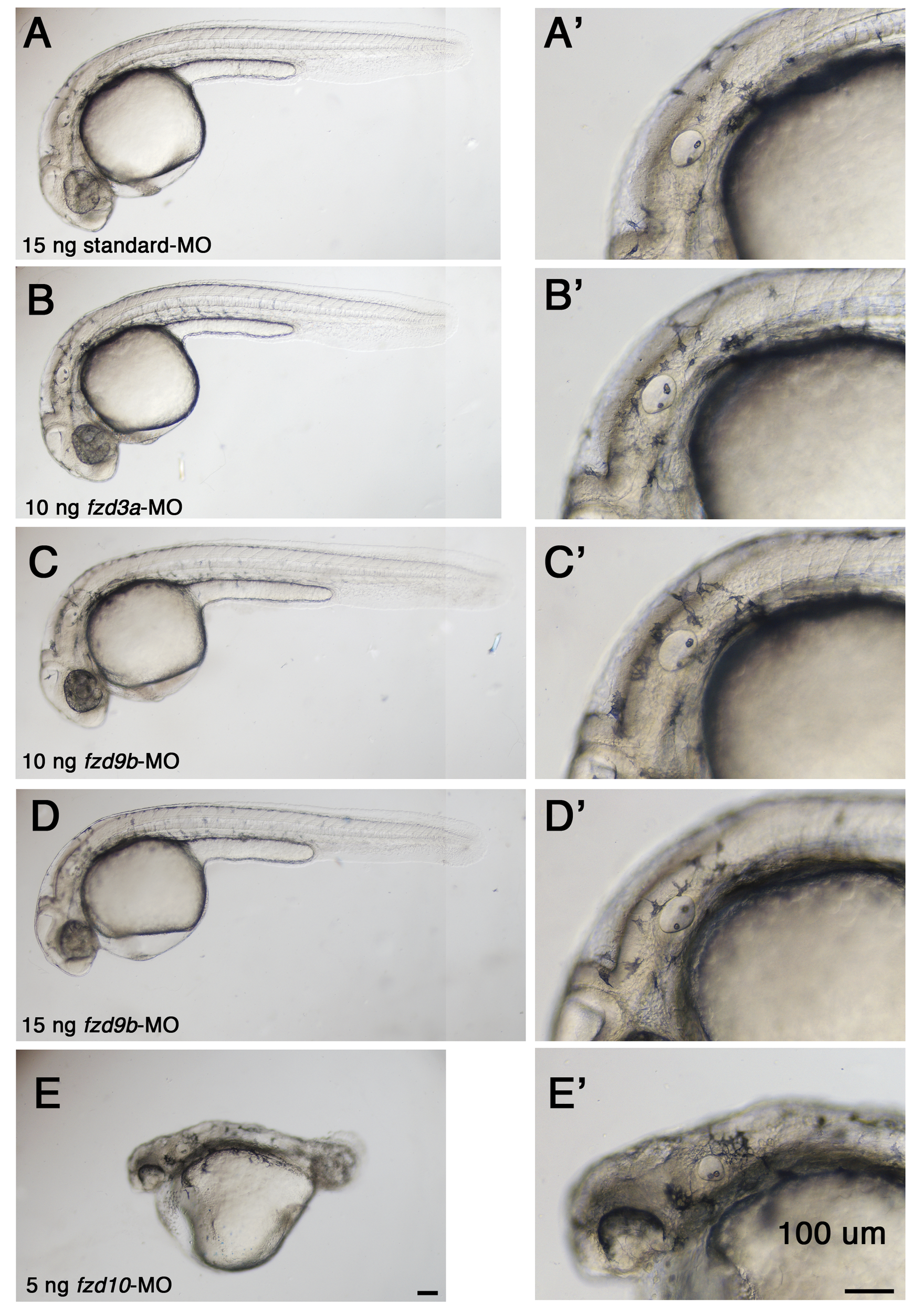

Supplement: Figure S4 — Phenotypes of embryos injected with fzd3a, fzd9b or fzd10 -MO alone. All are 32 hpf stages. Left side views with dorsal to the top. Name and the amount of each morpholino are shown in bottom left corner. A', B', C', D', E' are close up of the embryos shown in panels A, B, C, D, E, respectively. Scale bars: 100 µm. (TIF) [file pone.0054833.s004.tif]

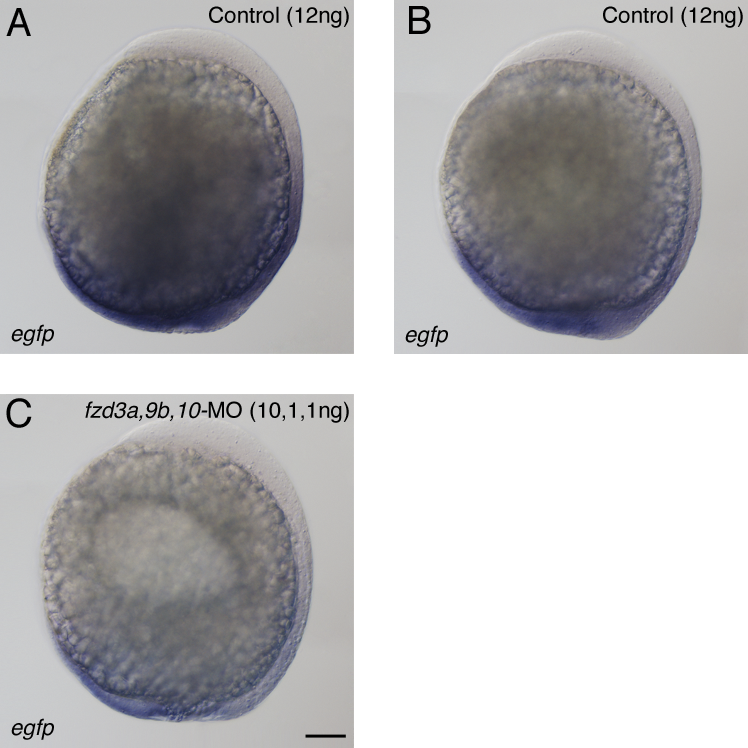

Supplement: Figure S5 — gfp expression in TopdGFP transgenic fish embryos injected with fzd -MOs. A, B) Typical expression patterns of control embryos categorised as “strong” (A) and “weak” (B) group. C) Embryo co-injected with 10 ng of fzd3a-MO, 1 ng of fzd9b-MO and 1 ng of fzd10-MO. This embryo was categorized as “weak” expression. Left side view with anterior oriented to the top. Bud stage. See Table S2 for quantitation. Scale bar: 100 µm. (TIF) [file pone.0054833.s005.tif]
