# Supplementary material for: A Systematic Survey of Expression and Function of Zebrafish frizzled Genes
Source: PLoS One. 2013 Jan 22;8(1):e54833. doi: 10.1371/journal.pone.0054833 (PMC3551900; doi:10.1371/journal.pone.0054833)
Supplement: Table S1 — Injection of single fzd-3a, 9b or 10 -MOs has no effect on melanophore formation. (DOCX) [file pone.0054833.s006.docx]

Supporting Table S1

Table S1. Injection of single *fzd-3a, 9b* or *10*-MOs has no effect on melanophore formation.

| Morpholinos | Amount | Straight body axis | | Abnormal (=kinked, shortened, no) body axis | | Dead | Total |
| --- | --- | --- | --- | --- | --- | --- | --- |
|  |  | Reduction of melanocytes | Normal melanisation |  |  |  |  |
| Random oligo | 5 ng | 0 | 403 | 11 | | 142 | 556 |
|  | 10 ng | 0 | 92 | 1 | | 96 | 189 |
| *fzd3a-MO* | 5 ng | 0 | 271 | 6 | | 45 | 322 |
|  | 10 ng | 0 | 208 | 1 | | 81 | 290 |
| *fzd9b-*MO | 5 ng | 0 | 97 | 0 | | 21 | 118 |
|  | 10 ng | 0 | 92 | 0 | | 51 | 143 |
| *fzd10-*MO | 5 ng | 0 | 31* | | 44 | 71 | 146 |
|  | 10 ng | 0 | 30 | | 47 | 74 | 151 |

Notes.

We did not examine the melanophore formation in embryos showing abnormal body axis because we supposed

melanophore formation might be affected by secondary defect of the general body malformation.

Observations were made at 3 dpf.

*One embryo shows different melanocyte pattern from wild type, though it still has plenty of those.
