# Supplementary material for: A Systematic Survey of Expression and Function of Zebrafish frizzled Genes
Source: PLoS One. 2013 Jan 22;8(1):e54833. doi: 10.1371/journal.pone.0054833 (PMC3551900; doi:10.1371/journal.pone.0054833)
Supplement: Table S2 — Combinatorial knock down of fzd-3a, 9b, 10 causes reporter down-regulation in TopdGFP transgenic fish. (DOCX) [file pone.0054833.s007.docx]

Supporting Table S2

Table S2. Combinatorial knock down of *fzd-3a, 9b, 10* causes reporter down-regulation in TopdGFP transgenic fish.

| Expression | Control (standard MO) | *fzd3a, 9b, 10*-MO |
| --- | --- | --- |
| strong | 11 (28) | 2 (5) |
| weak | 18 (46) | 23 (62) |
| no | 10 (26) | 12 (33) |
| total | 39 (100) | 37 (100) |

Notes.

Chi square test indicates the distribution of the three different expression types was significantly different

from the expected number based on Mendelian law in experimental group. On the other hand, proportion of three types of expression level in control was not significantly different from the expected value. Figures in parenthesis are percentages.
